# Increasing Care for Underserved Communities Through a Global Health Residency Training Program

**DOI:** 10.5334/aogh.4501

**Published:** 2024-11-22

**Authors:** Claire Zeigler, Zachary G. Jacobs, Sara U. Schwanke Khilji, MaryJoe K. Rice, Briana Frink, Patricia A. Carney

**Affiliations:** 1 Associate Professor of Medicine, Division of Hospital Medicine, Director for Global Health, Oregon Health & Science University, School of Medicine, Portland, OR, USA; 2 Clinical Educator, Portland Veterans Affairs Healthcare System, Portland, OR, USA; 3 Associate Professor of Medicine, Division of Hospital Medicine, Associate Director for Global Health, Oregon Health & Science University, School of Medicine, Portland, OR, USA; 4 Associate Professor of Medicine, Division of Hospital Medicine, Former Internal Medicine Site Director, Botswana Global Health Program, Oregon Health & Science University, School of Medicine, Portland, OR, USA; 5 Research Associate, Botswana-Harvard Health Partnership, Portland, OR, USA; 6 Internal Medicine Resident, Oregon Health & Science University, School of Medicine, Portland, OR, USA; 7 During the writing of this manuscript, Dr. Fink was a 4^th^ Year Medical Student at Oregon Health & Science University, now an intern in internal Medicine at the University of California San Diego, California, USA; 8 Professor of Family Medicine and of Internal Medicine, Division of Hospital Medicine, Oregon Health & Science University, School of Medicine, Portland, OR, USA

**Keywords:** Global Health, resident training, graduate medical education, physician workforce, underserved populations

## Abstract

*Background:* Global health education is important for addressing health inequities nationally and internationally. Physician shortages in underserved areas suggest more exposure during training is needed.

*Objective:* To study the impact of a global health training program on residents’ perceived preparedness and intention to care for underserved populations.

*Methods:* Observational mixed method evaluation of the impact of an educational intervention, the Global Health Scholars Program (GHSP), on perceived knowledge and intention to practice in underserved settings. The intervention consisted of a longitudinal global health training program addressing ethics, health equity, structural determinants of health, racism, colonialism, and systems-based practice. GHSP elective clinical rotations occurred at local underserved clinics, tribal and Indian Health Services (IHS) sites (Alaska, Arizona, Oregon), and in Botswana. A 16-item survey aligned with program objectives was administered to internal medicine residents at Oregon Health & Science University who completed the GHSP. This included five groups of residents who trained before coronavirus disease 2019 (COVID-19) (2016–2020) and three groups who trained during COVID-19 (2021–2023). Qualitative content analysis was conducted on open-ended text responses.

*Findings:* Surveys were sent to 45 participants; 37 responded (82.2%). All perceived knowledge variables increased significantly after training in the pre-COVID cohort. Among seven residents participating in GHSP during COVID, baseline scores were higher than in the pre-COVID cohort. Qualitative results indicate GHSP was a transformative educational experience and impactful on practice. Among current trainees, 42.9% reported moderate and 26.8% reported high/very high intention to practice in underserved settings. Among graduates, 40.9% reported practicing in underserved settings.

*Conclusions:* GHSP provides transformative educational experiences to residents, with knowledge gains on global health topics higher post-program compared with pre-program. Given 41% of participants in practice reported working in underserved settings, this intervention may help ameliorate physician workforce shortages.

## Introduction

Global health (GH) is “an area for study, research, and practice that places a priority on improving health and achieving equity in health for all people worldwide [1].” A cornerstone of GH is equity in both healthcare and social determinants of health, and so, by definition, GH is the act of caring for underserved populations everywhere in the world. The term *global* refers to the scope of health issues rather than geographic location, and thus is practiced locally as well as internationally. GH education within academic medicine in the United States of America (USA) focuses on teaching knowledge and skills to medical trainees that enable them to address equity in health across a range of contexts, and often specifically includes clinical training to promote health for vulnerable communities [1]. These efforts align with calls to address social determinants of health from many groups [2–4]. Concepts of global health training apply internationally and locally, given the persistent inequities in healthcare access and health outcomes across populations in the US and around the world [5, 6]. Within the US, some of these inequities are directly linked to workforce shortages of primary care providers, now reaching crisis levels [7–9].

Global health training is increasingly integrated into medical school and residency curricula [2–4, 10–15], though a 2017 systematic review found that certain specialties had higher percentages of residents participating in global health training compared with others [5]. High global health training specialties included preventive medicine (83%), emergency medicine (74%), and general surgery (71%) [5]. Notably absent from the top three specialties with global health training opportunities in the USA are the core primary care specialties: internal medicine (IM), family medicine, pediatrics, and obstetrics and gynecology. Multiple studies have demonstrated the positive association between global health training and intention to practice in underserved settings [16, 17]. Thus, we propose that incorporating global health education into IM residency training will better equip doctors to provide care to vulnerable populations both locally and internationally and potentially improve health outcomes.

We created an innovative global health longitudinal training program to provide IM residents with didactic education on global health topics and diverse clinical experiences. Based on the authors’ literature review, this is the first detailed description of a GH curriculum, taught asynchronously without extension of a standard 3‑year residency program and with associated outcomes related to knowledge, skills, and attitudes affecting intention to and actual practice of working in underserved settings. Formal didactics were complemented by practical training experiences in local Federally Qualified Health Centers (FQHCs), prisons/jails, the Indian Health Service and tribal health centers in Alaska, Arizona, California, and Oregon, and a public hospital in Botswana, located in Southern Africa. Here, we describe the impact of this program on residents’ perceived preparedness to care for underserved populations, and their intention to serve these populations after graduation.

## Methods

### Setting and residents

This study was conducted at Oregon Health & Science University (OHSU), a 576‑bed teaching hospital. The OHSU IM Residency Program comprises 109 residents on a 3+1 clinical schedule (3‑week inpatient, consults, or night float rotation and 1 week of outpatient continuity clinic). Residents have elective time during the second and third years of the postgraduate training program (PGY‑2 and PGY‑3).

### Global health scholars program (GHSP) description and participant selection

Established in 2014, the GHSP supplements the clinical and didactic training of the core IM program. It is offered to categorical residents interested in enhancing the standard IM residency training with care of underserved populations locally and internationally. Approximately ten interns apply per year, which represents approximately 33% of categorical interns.

**Figure 1:**
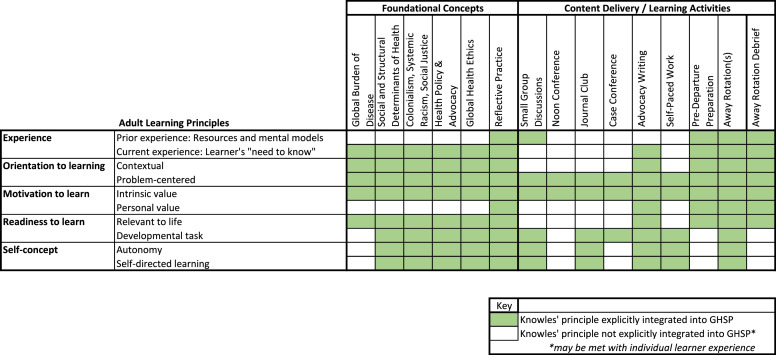
Integration of knowles’ adult learning principles into the GHSP curriculum. Source: Adapted from knowles, Holton & Swanson (1998), Androgogy in Practice.

The GHSP employs an adaptation of Knowles Adult Learning Theory using the following principles: self‑concept (autonomy and self‑directed learning), experience (encompassing both prior and current experience, the latter informing the “learner’s need to know”), orientation to learning (contextual and problem‑centered), motivation to learn (intrinsic and personal value), and readiness to learn (relevance to real‑life situations and incorporating developmental tasks) [18]. (Figure 1) These principles inform the GHSP at a number of levels, from the foundational concepts such as global health ethics and reflective practice to the array of educational settings through which content is delivered, including small group sessions, case discussions, self‑paced learning, and learner‑selected immersive clinical rotations. See Supplementary Table 1 for more details about the curriculum. The GHSP addresses topics related to the key curricular domains of social/structural competency, health equity, social and structural determinants of health, racism, colonialism, cost‑conscious care, ethics, and systems‑based practice. Many topics align with core competencies defined by the Accreditation Council for Graduate Medical Education [19]. Formal didactics include a resident‑led journal club every two months, eight noon conferences per year, plus additional context‑specific didactics during clinical rotations (Supplemental Table 1).

**Figure 2:**
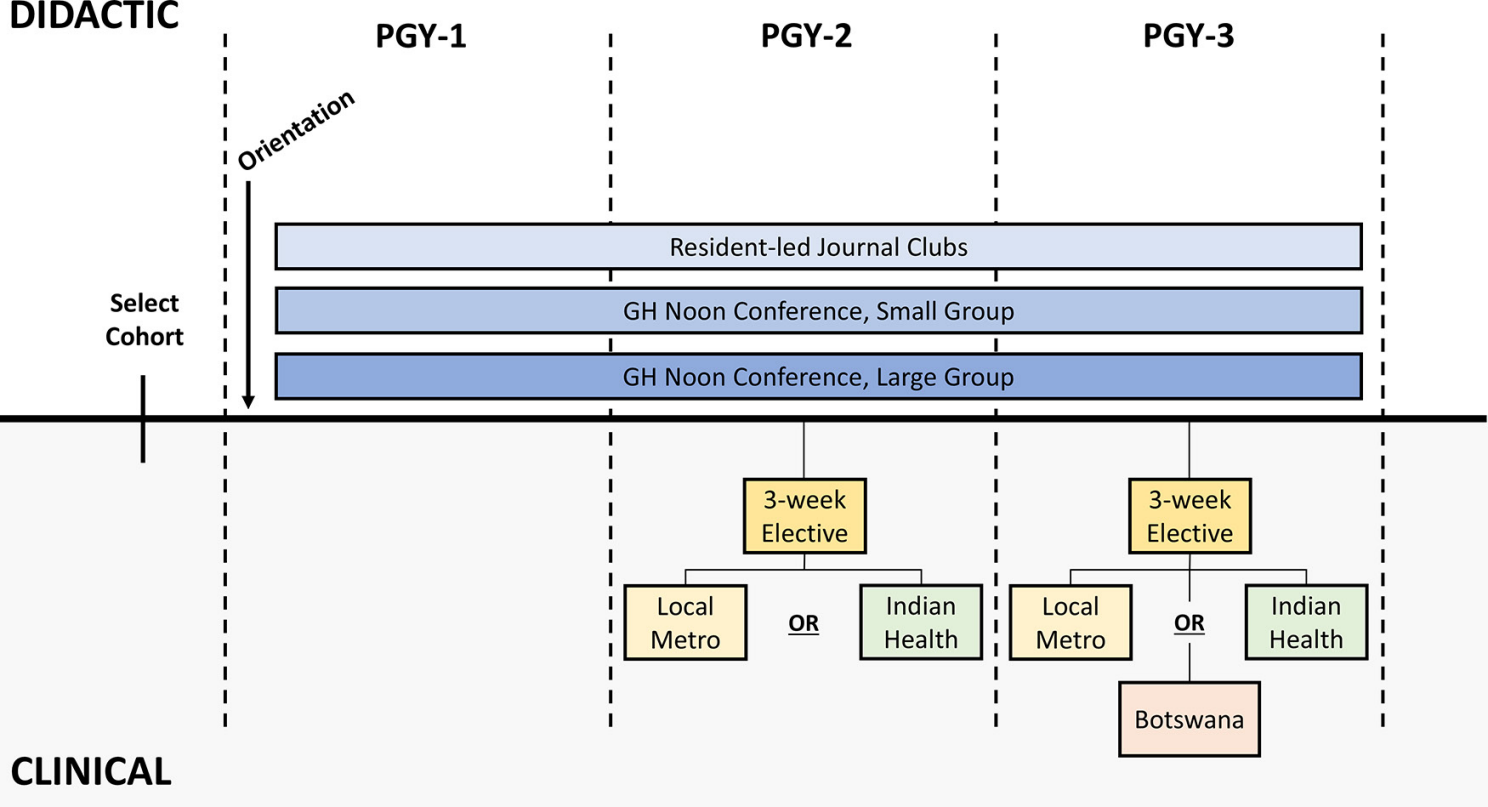
Overview of the GHSP curriculum over the three years of residency.

GHSP residents use their 3‑week elective time during PGY‑2 and PGY‑3 to complete global health clinical rotations (Figure 2). During PGY‑2, residents can choose to stay in the Portland area, where they work in underserved clinics and FQHCs that serve migrant and/or seasonal farmworkers and others with barriers to receiving healthcare. Alternatively, residents can choose to rotate at a site serving American Indian/Alaskan Natives, including the Alaska Native Medical Center in Anchorage, AK, or Indian Health Services (IHS) sites in Salem, OR, Tuba City, AZ, and Round Valley, CA. During PGY3, residents can complete a second rotation at one of the above sites. Alternatively, they may choose to rotate at Scottish Livingstone Hospital in Molepolole, Botswana, a longstanding international partner site where residents contribute to clinical education and quality improvement activities alongside an OHSU GHSP faculty member (author S.U.S.K) who lives and works in Botswana year‑round. Travel to these sites was notably disrupted by the coronavirus disease 2019 (COVID‑19) pandemic and residents in the GHSP during 2020–2022 completed only local (Portland area) rotations.

### Program evaluation and data capture

We designed a 16‑item survey mapped to program objectives that used a retrospective pre‑post design to assess participants’ understanding of topics taught as part of the program. This survey design is a more valid assessment of program impact on participant knowledge compared to typical pre‑post surveys, primarily because participants tend to overestimate their knowledge prior to participation and only recognize this after completing training (also known as response shift bias) [20–21]. Following completion of the GHSP, respondents assessed each survey variable by selecting a response to a five‑point Likert scale (1 = strongly disagree, 2 = disagree, 3 = neutral, 4 = agree, 5 = strongly agree) as it related to their understanding of the topics addressed *prior to participating* in the program and then *after program completion*. The survey also assessed demographics and intention to practice, or current practice, in underserved settings. If participants indicated low intention to practice in an underserved setting, they were asked to indicate a reason. Lastly, residents were asked what they liked best about the GHSP and what could be improved.

The survey was designed using a secure online platform (Qualtrics) and was pilot tested using cognitive interview techniques [22] prior to administration. The target population included IM residents who participated in the program between 2015 and 2022. Residents who participated in 2014 were excluded, as that was a pilot year followed by substantial programmatic changes. Invitations to complete the online survey were sent via e‑mail in March 2023. Two reminder emails were sent out one week apart. The Oregon Health and Science University institutional review board (IRB) reviewed study activities and determined it was not human subjects’ research (IRB #25182).

The survey was designed by authors C.Z., Z.G.J., and S.U.S.K. These same authors have been long‑standing GHSP faculty members. Learner interactions include engagement in diverse types of formal learning activities (small group sessions, journal clubs, reflective sessions, lectures) and clinical supervision. Authors P.A.C. and S.U.S.K. conducted thematic analyses of qualitative responses. Statistical analyses were conducted by author P.A.C., and all authors participated in the interpretation of both quantitative and quantitative data.

### Reflexivity statements

The author team comprises both medical learners, teaching faculty, and an educational researcher. Author C.Z. is a white, upper‑middle class, cisgender, straight, non‑disabled woman who spent most of her life living in Burundi, Colombia, the Philippines, and Mexico. She trained in IM with a focus on graduate medical education and has practiced primary care and hospital medicine at the Portland VA Hospital since 2014. She holds an appointment at OHSU as the Director of Global Health and recently stepped down as an Associate Program Director. She co‑created the GHSP in 2014. Author Z.G.J. is a white, upper‑middle class, cisgender, straight, non‑disabled man with training and experience in reflective practice and global health. He is the Associate Director of the GHSP and has experience working with Black, indigenous, and people of color (BIPOC) and underserved communities and used this lens to contribute to these analyses. Author S.U.S.K. trained in IM with a focus on Primary Care in the USA and holds a graduate degree in public health with emphasis on community health. She has lived and worked as an educator in Sri Lanka, Thailand, and Malaysia and has practiced as an Internist in Botswana since 2020. She provides site‑support for the GHSP program in Botswana and holds an adjunct teaching appointment at the University of Botswana, where she participates in clinical education activities. Author M.K.R. is a white, middle class, cisgender, non‑disabled woman who is currently in her third year of IM residency at OHSU and has participated in the GHSP throughout residency, including rotating at sites in Anchorage, AK, and Botswana. She plans to practice as a hospitalist after graduation and maintain involvement in GH education. Author B.F. is currently training in IM with a focus on Global Health, with continued medical education in Tijuana, Mexico and Maputo, Mozambique as a part of this training. She plans on practicing as a generalist in rural Brazil after the completion of residency. Author P.A.C. is a white, upper‑middle class, cisgender, straight, non‑disabled female with training and experience in both quantitative and qualitative (mixed methods) educational research. She was not directly involved in the development or implementation of the training program. She has conducted and published several qualitative mixed methods studies in educational research with BIPOC populations and used this lens to contribute to these analyses.

### Data analyses

Quantitative data were reviewed for completeness and frequencies were calculated for each variable. Categorical data were analyzed using chi‑squared test or Fishers’ exact test, where indicated. Means, standard deviations, and ranges in the pre‑test and post‑test periods were calculated and assessed using paired *t*‑tests. To further assess the educational meaningfulness of our findings, we calculated and presented effect sizes in our tables (Cohen’s *d* for *t*‑tests). A Cohen’s *d* of 0.50 or greater indicates a medium or educationally meaningful difference [23]. All tests were two‑tailed with alpha set at 0.05 to determine statistical significance (SPSS Version 29).

Free text responses were analyzed for emergent themes using classical content analysis [24]. Authors’ P.A.C. and S.U.S.K. independently coded the open text responses using a constant comparative approach to identify codes and then collapsed or expanded codes toward identify and then defining emergent themes. We then used consensus meetings to finalize and describe themes and select exemplars. As part of this work these authors voiced their positionality and checked in with each other about potential biases.

## Findings

Between 2015 and 2022, admission to the program included 10–13 interns per year, with a completion rate of 6–8 residents at graduation and a drop‑out of about 4–5 residents per class. The primary reasons for dropping out included residents wanting to use elective time for fellowship‑related research and inability to travel to Botswana during the pandemic.

The survey was sent to 45 participants and was returned by 37 for an 82.2% response rate. Most participants were between the ages of 31 and 35 years (67.6%) and identified as non‑Hispanic (97.3%) and white (89.2%) (Table 1). Seven were current residents or fellows; the majority trained between 2018 and 2023.

**Table 1:** Characteristics of study participants.

CHARACTERISTICS	VALUES *N* (%)
**Gender identity** Females Males Non‑binary Transgender Prefer to describe Prefer not to answer	(*n* = 37) 18 (48.6%) 19 (51.4%) 0 (0%) 0 (0%) 0 (0%) 0 (0%)
**Age category** 25–30 31–35 36–40	(*n* = 37) 2 (5.4%) 25 (67.6%) 10 (27.0%)
**Race** American Indian/Alaska Native Asian Black or African American Native Hawaiian or Pacific Islander White Other (please specify) Prefer not to answer	(*n* = 37) 4 (10.8%) 0 (0) 0 (0) 0 (0) 33 (89.2%) 0 (0) 0 (0)
**Ethnicity** Hispanic/Latinx Not Hispanic/Latinx Prefer not to answer	(*n* = 37) 1 (2.7%) 36 (97.3%) 0 (0%)
**Current resident/fellow** Yes No	(*n* = 29) 7 (24.1%) 22 (75.9%)
**Year Global Health Scholars Program completed** 2016 2017 2018 2019 2020 2021 2022 2023 (Still a resident)	(*n* = 37) 3 (8.1%) 1 (2.7%) 9 (24.3%) 8 (21.6%) 8 (21.6%) 4 (10.8%) 3 (8.1%) 1 (2.7%)

Perceived changes in knowledge before and after training are shown in Table 2. The pre‑training scores ranged from 2.43 for *Use of structural analysis to examine challenges faced by underserved populations* to 3.67 for both *Effects of physical and emotional health among those without housing* and *Critically reflect on clinical experiences during residency.* The post‑training scores ranged from 3.17 for *Use of structural analysis to examine challenges faced by underserved populations* to 4.40 for *Effects of inequities in healthcare on underserved communities* (Table 2). All 16 perceived knowledge variables increased statistically after training as compared to before training (*p* ≤ 0.007). The effect sizes ranged from 0.750 to 1.230, all of which were above the Cohen’s *d* threshold of 0.50 due to educationally meaningful differences.

**Table 2:** Changes in knowledge before and after participating in the Global Health Scholars Program.

ASSESSMENT VARIABLES ^C^ I UNDERSTOOD OR COULD…		MEAN (SD) BEFORE TRAINING (*N* = 30)	MEAN (SD) AFTER TRAINING (*N* = 30)	*P* VALUE^1^	EFFECT SIZE^2^
Effects of physical and emotional health among those without housing	*Range*	3.67 (0.66) *2–5*	4.27 (0.56) *3–5*	< 0.001	0.750
Health and access problems among immigrant populations	*Range*	3.20 (0.81) *2–4*	4.13 (0.78) *2–5*	< 0.001	0.828
Health‑related challenges among the prison population	*Range*	2.90 (0.71) *2–4*	3.33 (0.99) *2–5*	0.007	0.817
Health and well‑being challenges among those with human immunodeficiency virus (HIV)	*Range*	3.23 (0.73) *2–4*	4.10 (0.85) *2–5*	< 0.001	0.937
Health and access problems among indigenous populations	*Range*	2.87 (0.78) *2–5*	4.13 (0.82) *2–5*	< 0.001	0.868
Effects of *structural violence* on those living in underserved communities	*Range*	3.17 (0.99) *1–5*	4.03 (0.97) *2–5*	< 0.001	0.937
Effects of *inequities in healthcare* on underserved communities	*Range*	3.50 (0.82) *2–5*	4.40 (0.68) *3–5*	< 0.001	0.803
Effects of *systemic racism* on those in underserved communities	*Range*	3.20 (0.76) *2–5*	4.00 (0.83) *2–5*	< 0.001	0.887
Describe the context in which care is provided to Portland’s underserved communities	*Range*	2.57 (1.10) *1–5*	4.30 (0.60) *3–5*	< 0.001	1.230
Define structural violence and social determinants of health	*Range*	3.33 (0.96) *1–5*	4.30 (0.65) *3–5*	< 0.001	0.999
Describe how systemic racism impacts access to care and healthcare outcomes	*Range*	3.10 (0.89) *1–5*	4.07 (0.69) *3–5*	< 0.001	0.964
Use structural analysis to examine challenges faced by underserved populations	*Range*	2.43 (0.82) *1–4*	3.17 (1.10) *1–5*	< 0.001	1.048
Recognize the impact of historical trauma on communities today	*Range*	2.93 (0.94) *2–5*	3.97 (0.93) *2–5*	< 0.001	0.964
Identify potential structural solutions to improve care outcomes	*Range*	2.93 (0.69) *1–4*	3.83 (0.70) *3–5*	< 0.001	0.845
Critically reflect on clinical experiences during residency	*Range*	3.67 (0.55) *3–5*	4.30 (0.79) *1–5*	0.001	0.964
Engage in advocacy related to patient care	*Range*	3.07 (0.74) *2–4*	3.90 (0.92) *2–5*	< 0.001	0.791

Scale: 1 = strongly disagree; 2 = disagree; 3 = neutral; 4 = agree; 5 = strongly agree.

^1^Pre‑ versus post‑training, paired *t*‑test.

^2^Cohen’s *d* (a Cohen’s *d* of 0.50 or greater indicates a medium or educationally meaningful difference).

Among the seven current residents or fellows, three (42.9%) reported a moderate intention and two (26.8%) reported a high or very high intention to practice in underserved settings (Table 3). Nine (40.9%) GHSP graduates reported currently practicing in underserved settings, with most in urban underserved settings (Table 3). In addition, nearly all (90.0%) reported providing clinical services to patients on Medicaid, with houselessness, or with limited English proficiency in practice, mostly as hospitalists or specialists (Table 3). Whether training occurred before or during COVID‑19 did not appear to affect practice intentions or decisions (See Supplementary Tables).

**Table 3:** Intended or actual practice in underserved settings among global health scholar program graduates.

UNDERSERVED PRACTICE STATUS/PLANS	VALUES
**Recent graduates/residents (2021–2023)** *Intention to practice^1^* None/low Moderate High/very high	**(*n* = 7)** ***n* (%)** 2 (26.8%) 3 (42.9%) 2 (26.8%)
**If intention is none or low, reasons why** Family location decisions	**(*n* = 2)** 2 (100%)
**Graduates in independent practice** *Currently practice is in underserved setting:* Yes No	**(*n* = 22)** ***n* (%)** 9 (40.9%) 13 (59.1%)
**If no, reasons why^2^** Family location decisions Took hospitalist/academic medical center job	**(*n* = 11)** 6 (54.5%) 5 (45.5%)
**Graduates in independent practice^2^** *Current practice is in:* Urban underserved area Rural underserved area International underserved area	**(*n* = 4)** ***n* (%)** 3 (75.0%) 2 (50.0%) 1 (25.0%)
**Graduates in independent practice** *Current practice setting* Primary care (outpatient) Hospital medicine Specialty	**(*n* = 20)** ***n* (%)** 2 (10.0%) 12 (60.0%) 6 (30.0%)
**Populations regularly worked with^2^** FQHC Indigenous Houseless Corrections Migrant/immigrant Limited English proficiency LGBTQ+ Medicaid Veterans	**(*n* = 9)** 1 (11.1%) 4 (44.4%) 6 (66.7%) 2 (22.2%) 4 (44.4%) 5 (55.6%) 1 (11.1%) 7 (77.8%) 1 (11.1%)

^1^Scale: 1 = none; 2 = low; 3 = moderate; 4 = high; 5 = very high.

^2^Categories not mutually exclusive.

Supplementary materials show differences in demographic characteristics, scores, and intention to practice in underserved settings among those who trained before the COVID‑19 pandemic and those who trained during it, illustrating that the greatest increase in scores occurred among trainees in the pre‑COVID period (Supplementary Tables 2–4).

Complete qualitative findings are included in Supplementary Material Table 5. Briefly, five themes emerged when participants described the best aspects of the GHSP. The first was *Different learning setting and populations,* or the value of working with patients who were culturally, ethnically, and racially heterogeneous within diverse settings both inside and outside the USA. An exemplar for this theme is:

“Wonderful opportunity to learn in a completely different setting than primary site in Portland. Truly felt like I helped [an] underserved population.” *[Participant #3]*

The second emergent theme was *Transformative learning impacting personal and professional growth*, or the extent that the GHSP training program affected perceptions of the kind of physician participants wanted to be and how they approach clinical practice. An exemplar is:

“I grew more as a physician during my Botswana rotation than during any other part of my training. It also reminded me of why I went into medicine, kept me going, cured my burnout.” *[Participant #7]*

The third emergent theme was *Learning from each other*, which reflected an appreciation of what local clinicians, clinic teams, patients, and other residents taught them about patient care in underserved settings. An exemplar is:

“Opportunities to … interact with providers and patients in those settings full time. It’s one thing to read about the health challenges of a place such as Botswana, but it is quite another to understand firsthand what is happening there.” *[Participant #20]*

The fourth emergent theme was *Complementary learning experiences*, or an appreciation of learning opportunities that supplemented their education. Specific examples of cited learning opportunities included journal club, structured curriculum, online modules, and advocacy training. An exemplar is:

“I liked the monthly journal clubs in which we all got together and discussed a global health related topic or article. It was a great way to connect with fellow residents and explore health through a different lens.” *[Participant #21]*

The final emergent theme was *Social determinants of health focus,* or the perspective that the GHSP learning experiences broadened participants’ perspectives on inequities that affect the health and wellness of communities and how these factors might be mitigated by their efforts as physicians. An exemplar is:

“Working in a completely different inpatient and outpatient setting from our normal provided great insight to various barriers to health, downstream effects of systemic racism and cultural trauma.” *[Participant 11]*

Three themes emerged regarding what aspects of the GHSP could be improved. The first was *Better alignment between curriculum and clinical experiences*, or a desire for better structures for interactive learning and clinical practice experiences that more solidly aligned with global health concepts. Several respondents specified praxis opportunities that were novel to the learning context, such as advocacy. This was closely linked to a stated desire for more opportunities to apply these skills during clinical experiences. An exemplar for this theme is:

“More teaching on how to change things structurally such as through advocacy.” *[Participant #17]*

The second emergent theme was *Training site expansion*, or a desire for additional local and global clinical experiences with different populations, especially for US‑based sites and local marginalized communities. An exemplar is:

“I think it would be great if we connected more with the Portland community ‑ volunteering at food bank/shelters/medical clinics, etc. I think this would bolster the experiential learning of global health at a local level.” *[Participant #21]*

The final emergent theme was *Supplementary activities* that participants identified as those they thought would benefit program participants, including opportunities to interact with other learners and experts engaged in global health. An exemplar for this theme is:

“More training in how to be a clinician advocate in public health ‑ I feel well prepared to advocate for individual patients within the healthcare system but would have appreciated more experience in systems or community level advocacy.” *[Participant #27]*

## Discussion

We undertook this study to assess the extent to which a program such as the GHSP could address physician workforce issues in vulnerable communities. We found that 72% of residents or recent graduates of the program rated their intention to practice in underserved locations as moderate to very high, with nearly 41% of program graduates actively providing care in such communities. According to an Associatio of American Medical Colleges (AAMC) residency training report (2012–2021), only 24.1% of recent US residency graduates across all specialties reported working in a medically underserved area) [25]. This number is very similar for both graduates of IM (25.3%) and family medicine (26.1%) residencies, and in Oregon where 25.6% of all physicians practice in underserved areas [25]. Qualitative findings on the benefits of GHSP indicated that participants are indeed experiencing valuable clinical training in underserved locations. They appreciate both the diversity of the patients seen as well as the settings they are seen in. Many commented on how the experiences opened their eyes to the harms associated with health inequities and influenced their views on the physicians they wanted to be, as both advocates for patients and caregivers.

Transformative education, where deep fundamental shifts in world view occur related to educational experiences [26], certainly occurred here. Residents rotated in unfamiliar and often disorienting settings, which likely forced them to engage in introspection. A recent scoping literature review by Vipler et al. (2021) on transformative education in graduate medical education (GME) found that professional identity formation is often associated with such educational experiences [26]. Taken together, these findings suggest that both local and international GH experiences were educationally invaluable in triggering transformational learning.

To our knowledge, this is the first study to compare residents’ self‑reported knowledge, perceived preparedness, and intention to care for underserved populations following completion of an IM global health training program, as well as practice patterns post‑residency training. Our findings suggest the GHSP is significantly impactful in both training outcomes and career choices. In addition to assessing pre‑post differences, we also provided effect size values that further underscore the educational impact. Residents demonstrated changes in knowledge in care for houseless, indigenous, incarcerated, and immigrant patient populations and content related to structural racism, historical trauma, structural violence, and social determinants of health. Residents were more likely to work with underserved patient populations including the houseless, indigenous, immigrant, and resource‑poor (Medicaid) following graduation.

We felt it was not appropriate to ignore the global pandemic that occurred during the study period, and for this reason we included subanalysis in supplementary material. These data suggest the pandemic experience itself strongly affected awareness of the individual and collective responsibility for the well‑being of others and may explain why residents training during the COVID‑19 pandemic had higher baseline knowledge of global health matters. Importantly, independent of global events, the global health training program showed meaningful impacts on training outcomes and thus could be a key strategy to address physician shortages in underserved populations, including in primary care.

One area we would like to develop further is understanding how global health training empowers physicians in training and independent clinical practice to engage in advocacy. While important medical advances have occurred over the past decades, health disparities at national and global levels are overwhelming; increasing physician advocacy is an important professional activity to address these [27, 28]. Models for advocacy training in residency curricula have been illustrated by Peluso et al. [29], who developed a four‑session program on Advocacy and Activism with formal didactic teaching, training in basic skills, debate and discussion, and development and presentation of interprofessional advocacy projects. We plan to include more training in advocacy for future GHSP trainees and then assess how many of these trainees undertake subsequent advocacy work in their communities and beyond. Many of the other emergent themes regarding program improvements had been implemented prior to conducting this survey, with anticipated benefit to future cohorts.

Operating this program requires substantial programmatic resources and success is dependent on residents completing all requirements. To ensure commitment to the practice of global health, our program implemented a more formal selection process in July 2022, where residents were required to answer a series of short essay questions describing their interest in global health and how the program will help them achieve their goals. These are reviewed by the Associate and Assistant Program Directors to aid in GHSP participant selection. Furthermore, residents are no longer permitted to leave the program after enrolling, except in cases of extenuating circumstances. Moving forward, we plan to study the impact of these changes on attrition rate, changes in knowledge career choices, and resident satisfaction with the program.

The strengths of this study include achieving a response rate of 82%, which is considerably higher than other survey studies in residency education, which range from 3–50% [30]. Second, we utilized a retrospective pre‑post questionnaire design, which helps mitigate response shift bias that can lead a respondent to inaccurately assess their pre‑program knowledge or skills based on a dynamic understanding of the topic or question [20, 21]. Third, analysis of free text qualitative responses was performed independently by two separate investigators (S.U.S.K. and P.C.), with consensus meetings used to finalize these data.

Study limitations include that the outcomes were based on self‑report; thus, there were no objective measures of the program’s impact on residents’ knowledge or skills. That said, we are confident in the accuracy of the self‑reported data regarding practice in under‑resourced areas. In addition, respondents may have suffered from recall bias, particularly those who graduated several years prior to this study. Also, confounders related to time in independent practice could exist as well as other demographic characteristics, such as gender identity, race/ethnicity, and maturity and experience physicians attain in post‑residency practice could affect their perspectives. Another limitation was that we included a single residency training program, which limits generalizability, and the sample size was modest.

## Conclusion

The GHSP appears to provide transformative educational experiences to residents with direct impact on intention to practice and actual practice in underserved settings. Nearly three‑quarters of current trainees reported moderate‑to‑high intention to practice in an underserved setting and nearly 41% of graduates reported current work in underserved settings. These rates are significantly higher than those observed for graduates of US general internal medicine or family medicine residencies and those practicing in underserved areas in Oregon [25]. Many self‑reported knowledge gains on global health topics were statistically higher post‑program compared with pre‑program. Future research should focus on the impact of GH curricula on practice patterns across all medical specialties, as our research suggests this is a potential tool to address physician shortages in underserved communities.

## Figures and Tables

**Table:** 

GHSP	Global Health Scholars Program
OHSU	Oregon Health and Science University
FQHC	Federally Qualified Health Centers
IHS	Indian Health Services
GME	Graduate Medical Education
AAMC	Association of American Medical Colleges

## Data Availability

We will provide raw data upon request as long as a data use agreement is in place.

## Additional Files

The additional files for this article can be found as follows:

Supplementary File 1. Table 1. Discussion topics from the Global Health Scholars Program Curriculum during the 2022-2023 and 2023-2024 Academic Years.

Supplementary File 2. Table 2. Characteristics of study participants before and During COVID-19 pandemic.

Supplementary File 3. Table 3. Changes in knowledge before and after training according to when it occurred.

Supplementary File 4. Table 4. Intended or actual practice in underserved settings among Global Health Scholar Program graduates before and during the COVID-19 pandemic.

Supplementary File 5. Table 5. Qualitative findings for best aspects of OHSU’s Global Health Program and areas to be improved.
