# Supplementary material for: Increasing Care for Underserved Communities Through a Global Health Residency Training Program
Source: Ann Glob Health. 2024 Nov 22;90(1):70. doi: 10.5334/aogh.4501 (PMC11606394; doi:10.5334/aogh.4501)
Supplement: Supplementary File 1. — Table 1. Discussion topics from the Global Health Scholars Program Curriculum during the 2022-2023 and 2023-2024 Academic Years. [file agh-90-1-4501-s1.pdf]

Supplemental Table 1. Discussion topics from the Global Health Scholars Program Curriculum during the 2022-2023 and 2023-2024 Academic Years

	Discussion Topic
Large Group Didactics	Expanding health equity to accelerate an impactful public health response
	Building bridges to equity: amplifying community voices
	Ethics of a rock and a hard place
	Environmental justice and climate change
	American Indian/Alaska Native health and history
Small Group Didactics	Social and structural determinants of health and structural analysis
	Narrative medicine and reflective practice
	Global health as a medium for transformative learning
	Global burden of disease
	American Indian and Alaska Native health
	Point-of-care ultrasound for global health
Resident-led Journal club	Sustainable kidney care and climate change<br>Tuberculosis and HIV in Botswana
	Uranium mining in the Navajo Nation
	Value proposition for the new tuberculosis vaccine development
	Malaria and climate change
	Abortion medications in the Ukraine war
	Artificial intelligence in global health
