# Supplementary material for: Increasing Care for Underserved Communities Through a Global Health Residency Training Program
Source: Ann Glob Health. 2024 Nov 22;90(1):70. doi: 10.5334/aogh.4501 (PMC11606394; doi:10.5334/aogh.4501)
Supplement: Supplementary File 2. — Table 2. Characteristics of study participants before and During COVID-19 pandemic. [file agh-90-1-4501-s2.pdf]

Supplemental Table 2. Characteristics of Study Participants Before and During COVID-19 Pandemic

Characteristics	Totals n (%)	Before COVID- 19 2016-2020 n (%)	During COVID-19 2021-2023 n (%)	p value
**Gender Identity**	(n=37)	(n=29)	(n=8)	0.621†
Females	18 (48.6%)	14 (48.3%)	4 (50.0%)	
Males	19 (51.4%)	15 (51.7%)	4 (50.0%)	
Non-Binary	0 (0%)	0 (0%)	0 (0%)	
Transgender	0 (0%)	0 (0%)	0 (0%)	
Prefer to describe	0 (0%)	0 (0%)	0 (0%)	
Prefer not to answer	0 (0%)	0 (0%)	0 (0%)	
**Age Category**	(n=37)	(n=29)	(n=8)	**0.018***
25-30	2 (5.4%)	0 (0)	2 (25.0%)	
31-35	25 (67.6%)	20 (69.0%)	5 (62.5%)	
36-40	10 (27.0%)	9 (31.9%)	1 (12.5%)	
**Race**	(n=37)	(n=29)	(n=8)	0.640†
American Indian/Alaska Native	4 (10.8%)	3 (10.3%)	1 (12.5%)	
Asian	0 (0)	0 (0)	0 (0)	
Black or African American	0 (0)	0 (0)	0 (0)	
Native Hawaiian or Pacific Islander	0 (0)	0 (0)	0 (0)	
White	33 (89.2%)	26 (89.7%)	7 (87.5%)	
Other (Please specify)	0 (0)	0 (0)	0 (0)	
Prefer not to answer	0 (0)	0 (0)	0 (0)	
**Ethnicity**	(n=37)	(n=29)	(n=8)	0.784†
Hispanic/Latinx	1 (2.7%)	1 (32.4%)	0 (0)	
Not Hispanic/Latinx	36 (97.3%)	28 (96.6%)	8 (100.0%)	
Prefer not to answer	0 (0%)	0 (0%)	0 (0%)	
**Current Resident/Fellow**	(n=29)	(n=23)	(n=6)	0.131†
Yes	7 (24.1%)	4 (17.4%)	3 (50.0%)	
No	22 (75.9%)	19 (82.6%)	3 (50.0%)	
**Year Global Health Scholars Program Completed**	(n=37)	(n=29)	(n=8)	--
2016	3 (8.1%)	3 (10.3%)	--	
2017	1 (2.7%)	1 (3.4%)	--	
2018	9 (24.3%)	9 (31.0%)	--	
2019	8 (21.6%)	8 (27.6%)	--	
2020	8 (21.6%)	8 (27.6%)	--	
2021	4 (10.8%)	--	4 (50.0%)	
2022	3 (8.1%)	--	3 (37.5%)	
2023 (Still a Resident)	1 1 (2.7%)	--	1 (12.5%)	

†Fishers Exact Test

*Chi Square (p value excludes categories with missing data)
