# Supplementary material for: Increasing Care for Underserved Communities Through a Global Health Residency Training Program
Source: Ann Glob Health. 2024 Nov 22;90(1):70. doi: 10.5334/aogh.4501 (PMC11606394; doi:10.5334/aogh.4501)
Supplement: Supplementary File 3. — Table 3. Changes in knowledge before and after training according to when it occurred. [file agh-90-1-4501-s3.pdf]

Supplemental Table 3. Changes in Knowledge Before and After Training According to When it Occurred

	Before COVID-19[a]			*During COVID-19*[b]			
**Assessment Variables** [c]<br>**I understood or could…**	**Mean Before Training (n=23)**	**Mean After Training (n=23)**	**p value**[1]	**Mean Before Training (n=7)**	**Mean After Training (n=7)**	**p value**[1]	**pvalue**[2]
Effects of physical and emotional health among those without housing.	3.57 (0.66)	4.43 (0.59)	**<0.001**	4.00 (0.58)	4.14 (0.38)	0.356	0.141
Health and access problems among immigrant populations	3.09 (0.79)	4.17 (0.78)	**<0.001**	3.57 (0.79)	4.00 (0.82)	0.078	0.629
Health-related challenges among the prison population	2.87 (0.63)	3.43 (0.99)	**0.006**	3.00 (1.00)	3.00 (1.00)	--[d]	0.337
Health and well-being challenges among those with HIV	3.09 (0.73)	4.17 (0.94)	**<0.001**	3.71 (0.49)	3.86 (0.38)	0.356	0.202
Health and access problems among indigenous populations	2.74 (0.62)	4.14 (0.82)	**<0.001**	3.29 (1.11)	4.14 (0.90)	**0.017**	0.975
Effects of *structural violence* on those living in underserved communities	2.91 (0.95)	3.91 (1.04)	**<0.001**	4.00 (0.58)	4.43 (0.54)	0.078	0.097
Effects of *inequities in healthcare* on underserved communities	3.39 (0.84)	4.43 (0.66)	**<0.001**	3.86 (0.69)	4.29 (0.76)	0.078	0.650
Effects of *systemic racism* on those in underserved communities	3.09 (0.73)	4.04 (0.88)	**<0.001**	3.57 (0.79)	3.86 (0.69)	0.172	0.569
Describe the context in which care is provided to Portland's underserved communities	2.39 (0.99)	4.30 (0.64)	**<0.001**	3.14 (1.35)	4.29 (0.49)	**0.030**	0.936
Define structural violence and social determinants of health	3.13 (0.87)	4.26 (0.62)	**<0.001**	4.00 (1.00)	4.43 (0.79)	0.078	0.618

Describe how systemic racism impacts access to care and healthcare outcomes	2.96 (0.77)	4.00 (0.74)	**<0.001**	3.57 (1.13)	4.29 (0.49)	**0.047**	0.253
Use structural analysis to examine challenges faced by underserved populations	2.30 (0.77)	3.13 (1.14)	**0.002**	2.86 (0.90)	3.29 (1.1)	0.200	0.754
recognize the impact of historical trauma on communities today	2.74 (0.81)	3.87 (1.01)	<0.001	3.57 (1.13)	4.29 (0.49)	**0.047**	0.152
Identify potential structural solutions to improve care outcomes	2.87 (0.69)	3.87 (6.94)	<0.001	3.14 (0.69)	3.71 (0.76)	**0.030**	0.639
Critically reflect on clinical experiences during residency	3.61 (0.50)	4.43 (0.51)	<0.001	3.86 (0.69)	3.86 (1.35)	1.000	0.305
Engage in advocacy related to patient care	3.04 (0.71)	3.78 (0.95)	<0.001	3.14 (0.90)	4.29 (0.76)	**0.005**	0.173

[a]2016-2020 Combined

[b]2021-2023 Combined

[c] Scale – 1=Strongly Disagree; 2=Disagree; 3=Neutral; 4=Agree; 5=Strongly Agree

[d]t cannot be computed because the standard error of the difference is 0.

[1]Pre- vs Post Training, paired t-test

[2]Before- vs During COVID for post-test only, independent samples t-test
