# Supplementary material for: Increasing Care for Underserved Communities Through a Global Health Residency Training Program
Source: Ann Glob Health. 2024 Nov 22;90(1):70. doi: 10.5334/aogh.4501 (PMC11606394; doi:10.5334/aogh.4501)
Supplement: Supplementary File 4. — Table 4. Intended or actual practice in underserved settings among Global Health Scholar Program graduates before and during the COVID-19 pandemic. [file agh-90-1-4501-s4.pdf]

Supplemental Table 4. Intended or Actual Practice in Underserved Settings Among Global Health Scholar Program Graduates Before and During the COVID-19 Pandemic

Underserved Practice Status/Plans	Total	Before COVID-19 (2016-2020)	During COVID-19 (2021-2023)	p value†
**Recent Graduates/Residents (2021-2023)**	**(n=7)**	**(n=4)**	**(n=3)**	
Intention to practice[1]	**n (%)**	**n (%)**	**n (%)**	0.327
None/Low	2 (26.8%)	2 (50.0%)	0 (0)	
Moderate	3 (42.9%)	1 (25.0%)	2 (66.7%)	
High/Very High	2 (26.8%)	1 (25.0%)	1 (33.3%)	
**If intention is None or Low, Reasons Why**	**(n=2)**			
Family location decisions	2 (100%)	--	--	--
**Graduates in Independent Practice**	**(n=22)**	**(n=19)**	**(n=3)**	
Currently practice is in underserved	**n (%)**	**n (%)**	**n (%)**	0.544
setting:	9 (40.9%)	7 (36.8%)	2 (66.7%)	
Yes	13 (59.1%)	12 (36.2%)	1 (33.3%)	
No				
**If No, Reasons Why***	**(n=11)**			
Family location decisions	6 (54.5%)	--	--	--
Took hospitalist/academic medical center job	5 (45.5%)			
**Graduates in Independent Practice***	**(n=4)**	**(n=4)**	**(n=0)**	--
Current practice is in:	**n (%)**	**n (%)**	**n (%)**	
Urban Underserved Area	3 (75.0%)	3 (75.0%)	0 (0)	
Rural Underserved Area	2 (50.0%)	2 (50.0%)	0 (0)	
International Underserved Area	1 (25.0%)	1 (25.0%)	0 (0)	
**Graduates in Independent Practice**	**(n=20)**	**(n=17)**	**(n=3)**	
Current Practice Setting	**n (%)**	**n (%)**	**n (%)**	0.308
Primary Care (Outpatient)	2 (10.0%)	1 (5.9%)	1 (33.3%)	
Hospital Medicine	12 (60.0%)	11 (64.7%)	1 (33.3%)	

Specialty	6 (30.0%)	5 (29.4%)	1 (33.3%)	
**Populations Regularly Work With***	**(n=9)**	**(n=9)**	**--**	**--**
FQHC	1 (11.1%)	1 (11.1%)	--	
Indigenous	4 (44.4%)	4 (44.4%)	--	
Houseless	6 (66.7%)	6 (66.7%)	--	
Corrections	2 (22.2%)	2 (22.2%)	--	
Migrant/Immigrant	4 (44.4%)	4 (44.4%)	--	
Limited English Proficiency	5 (55.6%)	5 (55.6%)	--	
LGBTQ+	1 (11.1%)	1 (11.1%)	--	
Medicaid	7 (77.8%)	7 (77.8%)	--	
Veterans	1 (11.1%)	1 (11.1%)	--	

[1]Scale: 1=None; 2=Low; 3=Moderate; 4=High; 5=Very High

*Categories not mutually exclusive

†Chi-Square – cell sizes less than 5 do not produce stable results
