# Supplementary material for: Increasing Care for Underserved Communities Through a Global Health Residency Training Program
Source: Ann Glob Health. 2024 Nov 22;90(1):70. doi: 10.5334/aogh.4501 (PMC11606394; doi:10.5334/aogh.4501)
Supplement: Supplementary File 5. — Table 5. Qualitative findings for best aspects of OHSU’s Global Health Program and areas to be improved. [file agh-90-1-4501-s5.pdf]

Supplemental Table 5. Qualitative Findings for Best Aspects of OHSU's Global Health Program & Areas to be Improved

Assessment Category	Emergent Theme	Description	Exemplars
Best Aspects of the Program	Different learning setting & Population	The experience of working with patients who were culturally, ethnically, and racially different within a different setting both inside and outside the U.S., including beyond university hospital settings, was viewed as a valuable clinical educational experience.	• "Wonderful opportunity to learn in a completely different setting than primary site in Portland. Truly felt like I helped [an] underserved population." [Participant #3]<br>• "The Botswana clinical rotation was a great opportunity to learn about healthcare in a different setting with very different resources and helped me really consider how systems affect how healthcare is delivered." [Participant #4]<br>• "Having the opportunity to meet and care for so many indigenous people broadened my own understanding of the trials and tribulations faced by those people groups." [Participant #16]<br>• The clinical experiences at Chemawa were the most impactful, as this was a clinic that robustly serves Native American populations across the PNW. Virginia Garcia was also a valuable experience to better understand the underserved Hispanic population in Portland. [Participant #32]
	Transformative Learning Impacting Personal and Professional Growth	The extent to which the GHSP training program affected perceptions of the kind of physician participants wanted to be and how they approach clinical practice.	• "I grew more as a physician during my Botswana rotation than during any other part of my training. It also reminded me of why I went into medicine, kept me going, cured my burnout. [Participant #7]<br>• "Being able to spend time in Alaska and Botswana, which provided such an invaluable experience. It was the absolute best part of residency and really affected how I practice medicine to this day." [Participant #17]
	Learning from Each Other	Appreciation of what the local clinicians, clinic teams, patients, and other residents taught them about the care of patients in these underserved settings.	• "I really valued going to Botswana, I learned a great deal from the local providers, particularly as it relates to HIV care." [Participant #12]<br>• "Opportunities to … interact with providers and patients in those settings full time. It's one thing to read about the health challenges of a place such as Botswana, but it is quite another to understand firsthand what is happening there." [Participant #20]<br>• "I enjoyed working with the local house staff, meeting residents from other places in the US, and learning from the patient population" [Participant #6]

	Complementary Learning Experiences	Appreciation of learning opportunities that complemented experiential learning, including Journal Club, structured curriculum, online modules, advocacy training	• “I liked the monthly journal clubs in which we all got together and discussed a global health related topic or article. It was a great way to connect with fellow residents and explore health through a different lens. [Participant #21]<br>• “The faculty, the opportunities, the topics covered, the access to the University of Minnesota learning modules.” [Participant #13]<br>• “The local elective that involved learning how to execute an advocacy project.” [Participant #17]
	Social Determinants of Health Focus	Perspective that the GHSP learning experiences opened their eyes about inequities that affect the health and wellness of communities and how these factors might be mitigated by their efforts as physicians.	• “I really valued the … the focus on structural determinants of health. The program enabled me to evaluate structures, which [has] enabled me to build systems which create low barrier care.” [Participant #12]<br>• “Exposed us to the inequities of the underserved population and gave methods to help improve.” [Participant #17]<br>• “Working in a completely different inpatient and outpatient setting from our normal provided great insight to various barriers to health, downstream effects of systemic racism and cultural trauma.” [Participant # 11]
**Areas for Program Improvement**	Better Alignment Between Curriculum and Clinical Experiences	Desire for better structures for interactive learning and clinical practice experiences that more solidly aligned with global health concepts, especially those that are novel to learners, such as advocacy. This would allow for more opportunities to apply these skills during clinical experiences and more consistency amongst experiences.	• “More longitudinal experiences may have been interesting paired with more structured curriculum.” [Participant #4]<br>• “More formal didactic curricula around rural and global health issues would direct and focus the learning and interests of those in the program - this could be through webinars, a more robust group setting (beyond the journal club that was active during my time), or opportunities to network with others currently practicing in rural and global health and advocacy.” [Participant #37]<br>• More teaching on how to change things structurally such as through advocacy. [Participant #17]
	Training Site Expansion	Desire for more local and global clinical experiences to expand their experiences with different populations, with particular emphasis on	• “More exposure to diverse populations in Portland metro area.” [Participant #19]<br>• “More exposure to the African American population in Portland.” [Participant #17]

		additional US-based sites and local marginalized communities.	• “I think it would be great if we connected more with the Portland community - volunteering at food bank / shelters / medical clinics, etc. I think this would bolster the experiential learning of global health but at a local level.” [Participant #21]<br>• “More rural experience either through regional clinics in AK.” [Participant #37]<br>• “More global opportunities.” [Participant #6]
	Supplemental Activities	Participants readily identified activities that they thought would benefit program participants, including additional opportunities to interact with other learners and experts engaged in global health (both local and international)	• “Would be nice to have more dissemination of experiences/ideas after rotations among the others in the Global health track.” [Participant #17]<br>• “The clinical experience was great; I wish it had been accompanied (especially Alaska) with the opportunity for debrief afterwards. “[Participant #11]<br>• “Something to help improve Spanish language proficiency would be really nice, especially for people who end up practicing in Oregon.” [Participant #12]<br>• “Opportunities for research with the community sites.” [Participant # 22]<br>• “More integration of these ideas into the general internal medicine curriculum (everyone should work at project access!)” [Participant #31]<br>• “A more robust group setting (beyond the journal club that was active during my time), or opportunities to network with others currently practicing in rural and global health and advocacy.” [Participant #36]<br>• “More training in how to be a clinician advocate in public health - I feel well prepared to advocate for individual patients within the healthcare system but would have appreciated more experience in systems or community level advocacy.” [Participant #27]
